# Cancer-Associated Fibroblasts: Major Co-Conspirators in Tumor Development

**DOI:** 10.3390/cancers16010211

**Published:** 2024-01-02

**Authors:** Shubhangi Singh, Ajay P. Singh, Ranjana Mitra

**Affiliations:** 1 Department of International Studies (Global Health), College of Arts and Sciences, University of South Alabama, Mobile, AL 36688, USA; 2 Cancer Biology Program, Mitchell Cancer Institute, University of South Alabama, Mobile, AL 36604, USA; 3 Department of Pathology, Frederick P. Whiddon College of Medicine, University of South Alabama, Mobile, AL 36617, USA; 4 Department of Biochemistry and Molecular Biology, Frederick P. Whiddon College of Medicine, University of South Alabama, Mobile, AL 36688, USA; 5 Biomedical Sciences, College of Medicine, Roseman University of Health Sciences, Las Vegas, NV 89135, USA

The tumor microenvironment (TME) is a critical determinant of tumor progression, metastasis, and therapeutic outcomes. It consists of an extracellular matrix (ECM) and a variety of stromal cells, which remain in constant crosstalk with each other and the tumor cells present. Within this complex ecosystem, cancer-associated fibroblasts (CAFs) play a pivotal role, orchestrating the TME through intricate interactions with other cells, leading to the remodeling of the extracellular matrix (ECM) [1,2]. A recently published review article in the journal *Cancers* by Joshi et al. [2] marvelously captured the information related to the origin of CAFs and their multi-faceted roles in tumor development, metastasis, angiogenesis, the immune microenvironment, and therapeutic resistance. The authors also discuss the potential utility of CAFs as prognostic markers and therapeutic targets in types of different cancer.

The origin of CAFs is often resident quiescent fibroblasts; however, research also suggests that they can originate through the trans-differentiation of epithelial cells, endothelial cells, pericytes, smooth muscle cells, bone-marrow-derived mesenchymal cells, and adipocytes [3,4,5,6,7,8,9]. The proportion of CAFs derived from sources other than resident fibroblasts varies among different tumor types. For instance, in breast cancer, adipocytes contribute to CAFs, while in squamous cell carcinoma and pancreatic adenocarcinoma, pericytes and stellate cells, respectively, are the major contributors [6,8,9]. Interestingly, some reports also mention that CAFs can originate from cancer stem cells (CSCs) that gain myofibroblast-like features [10,11,12].

Characterizing CAFs has been challenging due to their considerable heterogeneity within tumors and across various cancer types [2,13]. Common markers used for CAF identification include alpha-smooth muscle actin (α-SMA), vimentin, fibroblast activation protein (FAP), fibroblast-specific protein 1 (FSP1), and platelet-derived growth factor receptor α/β (PDGFR-α/β) [2]. The diversity in these CAF markers often results from their trans-differentiation, influenced by growth factors, miRNAs, and exosomes secreted by cancer cells [9]. For example, factors such as CXCL12, Wnt7a, miR-9, and miR-125b from cancer cells, can prompt resident fibroblasts to transform into α-SMA-expressing CAFs [14]. Moreover, TGF-β and PDGF can induce resident fibroblasts to express FSP, while miR-370-3p can stimulate fibroblasts to secrete interleukin (IL)-1β, IL-6, and IL-8, contributing to their transition into CAFs. These secreted factors commonly activate pathways such as CXCR4/CXCL12, TGFβR/Smads, Wnt/β-catenin, and AKT/ERK during the transactivation process of CAFs. Elevated FAP and PDGFR-α/β levels in CAFs correlate with decreased survival rates, while heightened α-SMA expression is associated with intensified tumor growth and metastasis across multiple tumor types [15]. The diversity in CAF markers offers opportunities to utilize specific biomarkers and CAF density for predicting the nature of the disease and making prognostic assessments [16].

Once CAFs are established in the TME, they promote tumor growth through the secretion of growth factors, cytokines, and chemokines, as well as through the shedding of extracellular vesicles (EVs). CAF-secreted factors encompass IL6/8, IL1β, hepatocyte growth factor (HGF), TGF-β, stromal-derived factor-1 (SDF-1/CXCL12), and PDGF [2]. These factors activate distinct pathways to facilitate tumor growth across various tumor types. For instance, SDF-1/CXCL12 secreted by CAFs in pancreatic cancer promotes chemoresistance, making CXCR4 signaling an attractive target for therapeutic development [17,18]. CXCL12/CXCR4 signaling has also been shown to counteract docetaxel therapy in prostate cancer [19]. Similarly, osteopontin (OPN) and vascular cell adhesion molecule-1 (VCAM-1) secreted by CAFs in lung cancer activate AKT and MAPK signaling to promote tumor cell invasion and metastasis [20,21]. In breast cancer, α-SMA-positive CAFs induce EMT in tumor cells by secreting TGF-β and CXCL12 to activate their downstream signaling [22,23]. Similarly, CAFs from colorectal and ovarian cancers are shown to secrete FGF-1, which stimulates MAPK/ERK signaling and promotes aggressive tumor phenotypes [24,25].

CAFs’ role in tumor invasion and metastasis involves orchestrating ECM remodeling and triggering EMT and actin-rich invadosome formation in tumor cells. In addition, through dysregulation of enzymes like lysyl oxidases (LOXs), matrix metalloproteases (MMPs), and transglutaminases, CAFs disrupt ECM stability, fostering invasion and dissemination of cancer cells [13,26]. Activation of TWIST, ZEB, SNAIL/SLUG transcription factors by CAFs induces EMT, leading to heightened mesenchymal markers (vimentin, fibronectin, N-cadherin) and suppression of epithelial junction proteins (E-cadherin, occludins, claudins) [27,28]. CAF-secreted TGF-β crucially promotes EMT, enhancing markers such as vimentin, SNAIL, and ZEB2 [29,30]. Moreover, CAFs contribute to EMT through the non-canonical Wnt pathway, often interacting with STAT-3 and survivin [31]. Other factors such as IL-6, IL-8, OPN, HGF, and CXCL12 also influence EMT through diverse pathways. For example, OPN affects the TWIST pathway, CXCL-12 triggers Wnt/β-catenin, HGF facilitates collagen degradation, and IL-6 activates the STAT3 and MEK/ERK pathways [32,33,34]. The variations in cytokine secretion among CAFs in different tumors add complexity to understanding their specific EMT-promoting mechanisms.

Emerging evidence suggests that CAFs frequently utilize exosomes to communicate with tumor cells. Additionally, these exosomes carry various microRNAs (miRNAs), facilitating migration, invasion, metastasis, and therapeutic resistance. Among the most prevalent miRNAs found in CAF exosomes across different tumors are miR-21, miR378-e, miR-148, and miR-92a-3p, known to be involved in EMT and therapeutic resistance mechanisms [35].

It is suggested that cancer cells rely predominantly on glycolysis and not on mitochondrial oxidative phosphorylation (OXPHOS) for their energy needs even in the presence of oxygen, a process known as aerobic glycolysis or the “Warburg effect”. However, in some cancer cells, a “Reverse Warburg effect” is noted which results from the metabolic coupling between cancer and stromal cells. Neoplastic cells trigger oxidative stress in nearby fibroblasts by releasing reactive oxygen species (ROS), prompting glycolysis and lactate production through NF-κB signaling. This reprograms CAFs, lowering caveolin 1 (CAV1) and increasing monocarboxylate transporter 4 (MCT4), leading to lactate release. Nearby cancer cells respond with heightened MCT1, enabling lactate uptake and T53-induced glycolysis and apoptosis regulator (TIGAR) which suppresses glycolysis and increases OXPHOS [36,37]. Similar observations have also been made between hypoxic and non-hypoxic cancer cells [38]. In other instances, CAFs are shown to reuse cancer-derived lactate to maintain fibrotic conditions and induce immunosuppression [39]. Thus, the metabolic partnership between cancer cells and CAFs enables the efficient use of available nutrient resources and oxygen to favor uninhabited tumor growth and metastasis.

The intricate involvement of CAFs in tumor growth, invasion, and metastasis positions them as potential therapeutic targets. Various markers expressed in CAFs and pathways crucial to tumor growth and metastasis have been the focus of targeted therapies in both pre-clinical and clinical trials. For instance, targeting the TGF-β pathway in hepatic, ovarian, pancreatic, and breast cancers has shown efficacy in preventing CAF activation and inhibiting metastasis [40,41,42,43]. However, clinical trials aimed at inhibiting MMPs did not yield the desired outcomes, although monoclonal antibody targeting demonstrated a reduction in tumor growth and metastasis [44]. Similarly, targeting FAP with antibodies in lung cancer has shown promise, but its non-specific expression in CAFs is associated with potential side effects [16,45]. Furthermore, targeting SDF-1, TGF-β, and IL-6 in CAFs has displayed the potential to reverse tumor immunosuppression in clinical trials [46]. In pancreatic and breast cancers, targeting the hedgehog pathway exhibited potential by reducing fibroblast accumulation [47] but was met with failure in a later clinical trial [48]. Similarly, in another study, the depletion of CAFs induced immunosuppression and promoted tumor aggressiveness [49]. Thus, CAFs’ involvement in cancer progression is more complex than thought and may vary along the course of cancer evolution. The complexity arising from diverse CAF subpopulations may underlie such contrasting observations and must be studied in detail in order to develop a clinically useful intervention approach. Thus, our comprehension of CAF markers, subpopulations, and signaling is still incomplete and requires extensive investigations to reap clinical benefits for cancer patients from their therapeutic targeting.
